# Pulmonary Hypertension in Children is Associated With Abnormal Flow Patterns in the Main Pulmonary Artery as Demonstrated by Blood Speckle Tracking

**DOI:** 10.1016/j.cjcpc.2022.09.001

**Published:** 2022-09-09

**Authors:** Wadi Mawad, Solveig Fadnes, Lasse Løvstakken, Matthew Henry, Luc Mertens, Siri Ann Nyrnes

**Affiliations:** a Division of Cardiology, Department of Paediatric, The Hospital for Sick Children, University of Toronto, Toronto, Ontario, Canada; b Department of Circulation and Medical Imaging, Norwegian University of Science and Technology (NTNU), Trondheim, Norway; c Department of Paediatrics, Montreal Children’s Hospital, McGill University Health Centre, Montréal, Québec, Canada; d Moere & Romsdal Hospital Trust, Division of Aalesund Hospital, Department of Pediatrics, Aalesund, Norway; e Children’s Clinic, St. Olavs University Hospital, Trondheim, Norway

## Abstract

**Background:**

Paediatric pulmonary arterial hypertension (PAH) is characterized by increased pulmonary vascular resistance resulting in increased pulmonary artery (PA) and right ventricular pressure (RV). This is associated with disturbed flow dynamics in the PA and RV that are not well characterized. We aimed to compare flow dynamics in children with PAH compared with healthy controls using blood speckle tracking echocardiography.

**Methods:**

Patients <10 years of age with PAH and healthy controls were included. We examined flow dynamics in the main PA (MPA) and right ventricle based on acquisition blood speckle tracking images obtained from the RV and PA. Qualitative and quantitative analyses were performed.

**Results:**

Eighteen subjects were included in each group. A diastolic vortex in the MPA was identified in 16 of the patients with PAH, but not in controls. Significantly higher MPA systolic (4.84 vs 2.42 mW/m; *P* = 0.01) and diastolic (0.69 vs 0.14 mW/m; *P* = 0.01) energy loss, as well as increased vector complexity (systole: 0.21 vs 0.04, *P* = 0.003; diastole: 0.13 vs 0.05, *P* = 0.04) and diastolic vorticity (15.2 vs 4.4 Hz; *P* = 0.001), were noted in PAH compared with controls.

**Conclusion:**

This study demonstrates the presence of abnormal flow patterns in the MPA with diastolic vortex formation in most patients with PAH. This diastolic vortex likely results from reflected waves from the distal pulmonary bed. Our data indicate that the diastolic vortex could potentially be used in the diagnosis of PAH. The clinical significance of the energy loss findings warrants further investigation in a larger cohort of patients with PAH.

## Résumé

**Contexte:**

L’hypertension artérielle pulmonaire (HTAP) pédiatrique est caractérisée par une résistance vasculaire pulmonaire accrue qui donne lieu à une augmentation de la pression dans l’artère pulmonaire (AP) et dans le ventricule droit (VD). Ce phénomène s’accompagne de perturbations de la dynamique des débits dans l’AP et le VD, qui n’ont pas encore été bien caractérisées. Nous avons cherché à comparer la dynamique des débits chez des enfants atteints d’HTAP avec celle de témoins en bonne santé en utilisant l’échocardiographie de suivi des marqueurs acoustiques du sang.

**Méthodologie:**

Des patients de moins de 10 ans atteints d’HTAP et des témoins en bonne santé ont participé à l’étude. La dynamique des débits du tronc pulmonaire (TP) et du ventricule droit a été examinée à partir d’images de suivi des marqueurs acoustiques du sang de l’AP et du VD. Des analyses qualitatives et quantitatives ont aussi été réalisées.

**Résultats:**

Dix-huit sujets ont été inclus dans chacun des groupes. Un vortex diastolique du TP a été observé chez 16 des patients atteints d’HTAP, mais n’était présent chez aucun des témoins. Une perte d’énergie significativement plus élevée dans le TP a été notée pour la systole (4,84 *vs* 2,42 mW/m; *P* = 0,01) et la diastole (0,69 *vs* 0,14 mW/m; *P* = 0,01) des patients atteints d’HTAP; de plus, une complexité vectorielle accrue (systole : 0,21 *vs* 0,04, *P* = 0,003; diastole : 0,13 *vs* 0,05, *P* = 0,04) et une vorticité diastolique accrue (15,2 *vs* 4,4 Hz; *P* = 0,001) ont été notées chez les patients atteints d’HTAP comparativement aux témoins.

**Conclusion:**

Notre étude fait état d’un profil circulatoire anormal caractérisé par la formation d’un vortex diastolique dans le TP chez la plupart des patients atteints d’HTAP. Ce vortex découle probablement d’ondes réfléchies du lit pulmonaire distal. Les données que nous avons obtenues indiquent que le vortex diastolique pourrait possiblement être utilisé dans le diagnostic de l’HTAP. Par contre, la signification clinique des résultats concernant la perte d’énergie nécessite d’autres études auprès d’une cohorte plus importante de patients atteints d’HTAP.

Paediatric pulmonary artery hypertension (PAH) is defined by increased pulmonary artery pressure and pulmonary vascular resistance (PVR) resulting in increased right ventricular pressure loading. The impact of PAH on right ventricular (RV) morphologic and functional parameters has been extensively studied, whereas there is limited data available on the changes in flow dynamics associated with PAH.^1^, ^2^, ^3^ Four-dimensional flow magnetic resonance imaging (4D-flow MRI) has been used for qualitative and quantitative flow assessment in PAH. Flow characteristics were shown to differ significantly between patients with PAH and healthy controls (CTL).^4^ A decrease in peak systolic vorticity (VO) in the main (MPA) and right pulmonary artery (RPA) was associated with increased PVR and was used in a model to noninvasively predict PVR.^5^ Although 3-dimensional assessment of flow is a major advantage of 4D-flow MRI, it comes at the expense of low temporal resolution. This limitation is more significant in children as they have higher heart rates. Blood speckle tracking (BST) echocardiography is a 2-dimensional technique that allows blood flow visualization at high temporal resolution.^6^ This allows the study of more short-lived flow events such as early diastolic vortex formation in the MPA. The aim of the current study was to use BST to (1) qualitatively describe MPA flow patterns in patients with PAH compared with healthy CTL and (2) compare quantitative flow parameters including energy loss (EL), VO, and vector complexity (VC) in the MPA and RV between patients with PAH and CTL. The relationship between flow parameters, RV functional parameters, and RPA distensibility (RPA_D_) will be evaluated.

## Materials and Methods

**Figure 1:**
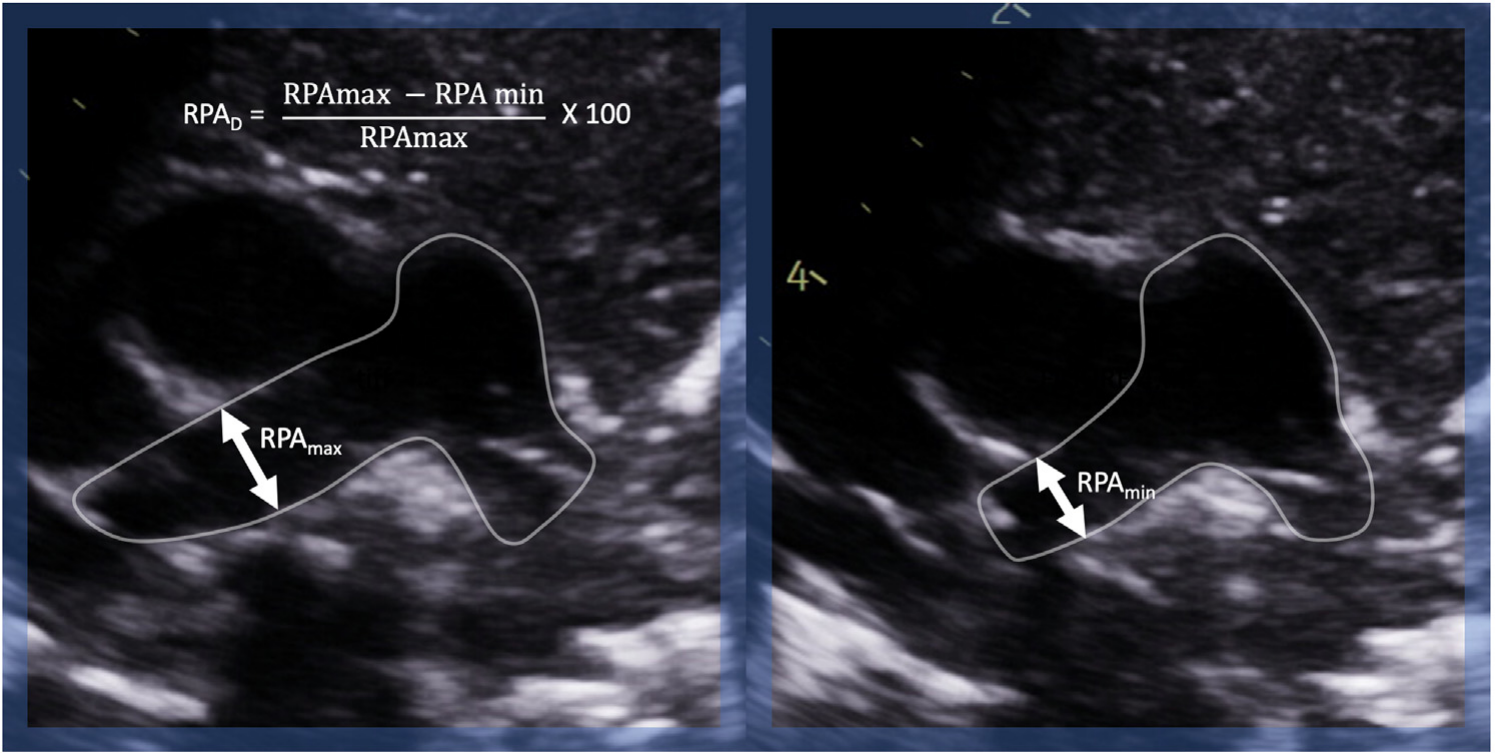
Right pulmonary artery distensibility (RPA_D_) index measurement in a parasternal short-axis view of the pulmonary arteries. max, maximal measurement; min, minimum measurement.

Between December 2015 and December 2020, we included patients from the Hospital for Sick Children in Toronto, Canada, and St. Olav’s Hospital in Trondheim, Norway, who were <10 years of age and who had PAH as defined by an estimated mean pulmonary artery pressure measured by echocardiography exceeding 25 mm Hg as well as healthy CTL matched for age and sex. The study was approved by both institutions’ ethics review boards, and informed consent was obtained before enrolment. A Vivid E9 or E95 system (GE Vingmed Ultrasound, Horten, Norway) with research software enabling image acquisitions with a frame rate equal to the pulse repetition frequency, that is, in the kHz range, was combined with the B-mode modality. We acquired a short-axis view of the MPA and RV centred apical views. At least 2 cardiac cycles were recorded with commercially available ultrasound probes including the 6S and 12S phased-array probes (GE Healthcare, Milwaukee, WI). Storage of IQ (in-phase and quadrature) data was done to enable full offline postprocessing and BST analysis. Dedicated in-house software analysis tools were used (PyUSview; NTNU, Trondheim, Norway). Temporal and spatial smoothing parameters were kept identical for all analyses (temporal smoothing = 40 milliseconds, Gaussian spatial smoothing = 5 × 5 mm^2^). The MPA flow field and RV were segmented, and VO, EL, and vector complexity were quantified in the MPA and RV. The calculation of EL and its validation and variability have been previously described by our group.^7^^,^^8^ The definition of VO as calculated based on BST has been previously published.^7^ Vector complexity has been described using other methods.^9^ This parameter describes the flow complexity by quantifying the spread of the velocity vectors. It is defined as 1 − r, where r is the vector concentration as defined by Pedersen et al.^10^ The measurements range from 0, where all velocity vectors are in the same direction, to 1, indicating vectors in multiple different directions. It can be thought of as a measure of flow laminarity, with laminar flow having vector complexity approaching 0 and turbulent flow approaching 1. Conventional echocardiographic parameters were obtained based on standard clinical methodology. RPA distensibility was measured as the percentage difference between the maximal and minimal RPA diameters in a parasternal short-axis view (Fig. 1).

### Statistical analysis

Comparison between 2 groups was done using the Mann-Whitney *U* test with a *P* value of <0.05, which was considered statistically significant. Correlations were tested using Spearman’s rank test. The analyses were conducted using GraphPad Prism 8 (GraphPad Software, La Jolla, CA).

## Results

Demographic and hemodynamic characteristics are summarized in Table 1. In total 36 subjects were included, 18 patients with PAH and 18 CTL. All patients with PAH had idiopathic PAH and no history of previous cardiac surgery. The majority (13 of 18) were receiving antipulmonary hypertensive therapy (sildenafil: 4; sildenafil + oxygen: 8; sildenafil + bosentan + macitentan + selexipag: 1). There were no significant differences in baseline characteristics between the groups. The PAH group had a larger MPA size and lower tricuspid annular systolic excursion and right ventricular fractional area change.

**Table 1:** Demographic and echocardiographic data

	PAH (n = 18)	CTL (n = 18)
Male (%)	50	44
Age (y)	2.8 (0.5-4.2)	3.2 (0.5-4.8)
Height (m)	110 (89-125)	99 (80-122)
Weight (kg)	18.9 (15.4-32.3)	15.8 (10.9-25.6)
BSA (m^2^)	0.75 (0.65-0.81)	0.66 (0.50-0.94)
HR (BPM)	86 (75-108)	97 (81-110)
MPA z-score	2.77 (1.62-3.26)	−0.20 (−1.74 to 1.01)∗
RPA distensibility (%)	23.9 (12.7-31.7)	24.5 (17.2-30.4)
RV systolic pressure (mm Hg)	82 (42-105)	
RV systolic pressure (% of systemic)	79 (46-104)	
TAPSE z-score	−1.30 (−3.83 to 6.80)	0.02 (−1.18 to 4.71)∗
RVFAC (%)	33.5 (30-38)	37 (36-40)∗
MPA		
Diastolic MPA vortex	16/18	0/18
MPA diastolic vortex duration (ms)	150 (121-171)	
Avg. VC_S_	0.21 (0.08-0.42)	0.04 (0.02-0.06)∗
Avg. VC_D_	0.13 (0.06-0.30)	0.05 (0.02-0.16)∗
Avg. EL_S_ (mW/m)	4.84 (2.16-11.67)	2.42 (1.11-3.86)∗
Avg. EL_D_ (mW/m)	0.69 (0.28-2.17)	0.14 (0.03-0.39)∗
Avg. VO_S_ (Hz)	17.1 (15.8-21.6)	27.7 (20.6-35.1)
Avg. VO_D_ (Hz)	15.2 (11.1-20.9)	4.4 (0.2-8.4)∗
RV		
Avg. VC_S_	0.27 (0.12-0.42)	0.13 (0.09-0.28)∗
Avg. VC_D_	0.27 (0.15-0.34)	0.12 (0.09-0.16)∗
Avg. EL_S_ (mW/m)	1.54 (0.32-2.67)	0.68 (0.25-1.12)
Avg. EL_D_ (mW/m)	4.08 (2.19-7.20)	2.87 (1.44-4.86)
Avg. VO_S_ (Hz)	22.8 (17.9-27.3)	22.1 (18.3-27.5)
Avg. VO_D_ (Hz)	25.1 (18.5-31.3)	24.2 (17.8-28.2)

BSA, body surface area; BPM, beats per minute; CTL, controls; EL_D_, diastolic energy loss; EL_S_, systolic energy loss; FAC, fractional area change; HR, heart rate; MPA, main pulmonary artery; PAH, pulmonary arterial hypertension; RPA, right pulmonary artery; RV, right ventricle; RVFAC, right ventricular fractional area change; TAPSE; tricuspid annular plane systolic excursion; VC_D_, diastolic vector complexity; VC_S_, systolic vector complexity; VO_D_, diastolic vorticity; VO_S_, systolic vorticity.

∗ When *P* value <0.05; values expressed as median, quartiles (Q1-Q3).

**Figure 2:**
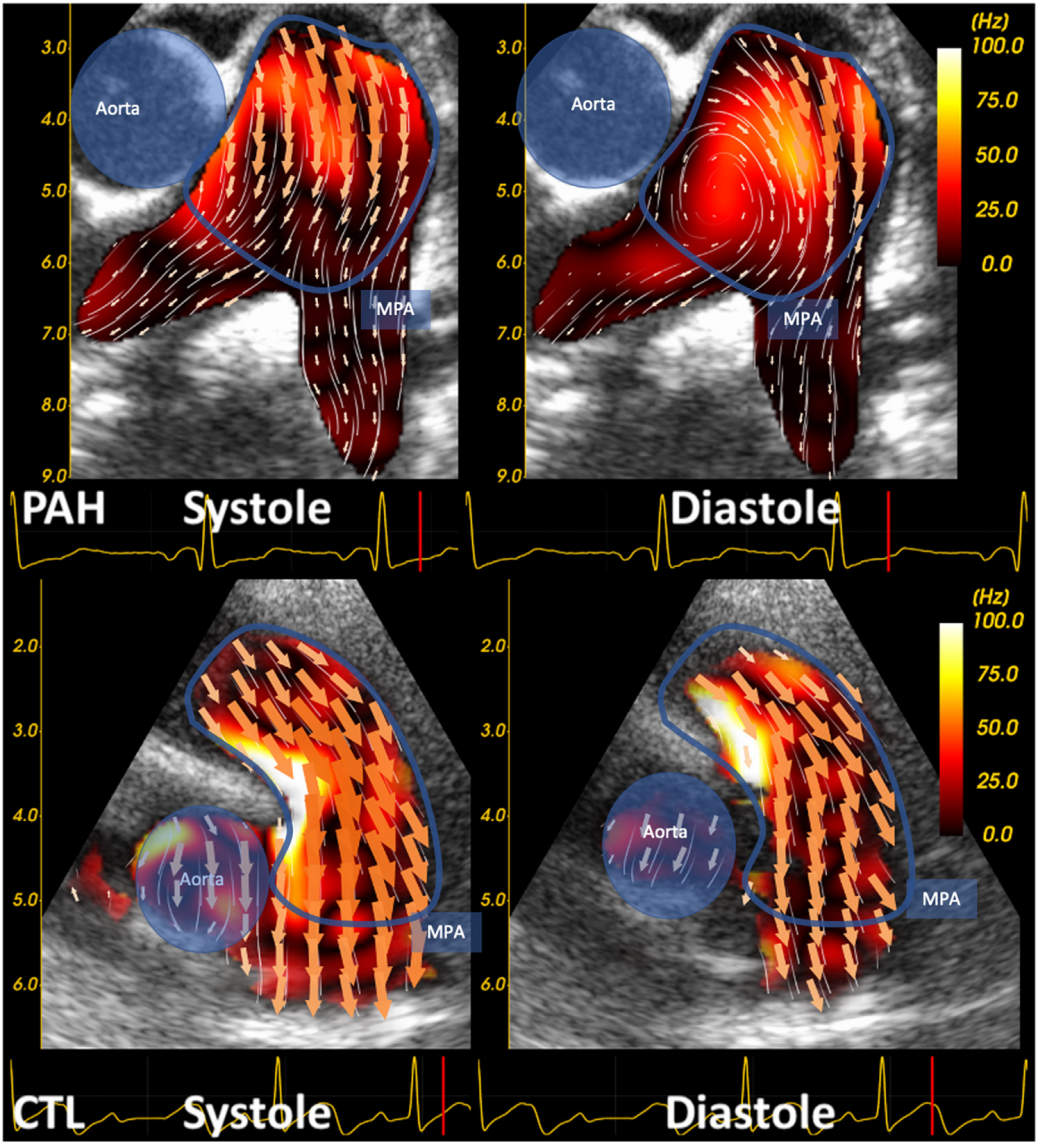
Typical main pulmonary artery (MPA) flow patterns as shown by high-frame-rate ultrasound imaging and blood speckle tracking. The vorticity map is seen in the background. CTL, control; PAH, pulmonary arterial hypertension.

Qualitatively, both groups had laminar flow in the MPA in systole. A diastolic vortex in the MPA was observed in 16 of 18 patients with PAH, whereas it was not present in any of the CTL. The diastolic vortex was first observed in the RPA origin in 6 of 16 patients with PAH and in the MPA in 10 of 16 patients. All vortices had a clockwise rotation and dissipated as they migrated from their origin towards the pulmonary valve. In CTL diastolic flow remains laminar without any detectable rotation. Figure 2 shows typical examples from each group illustrating the flow patterns.

**Figure 3:**
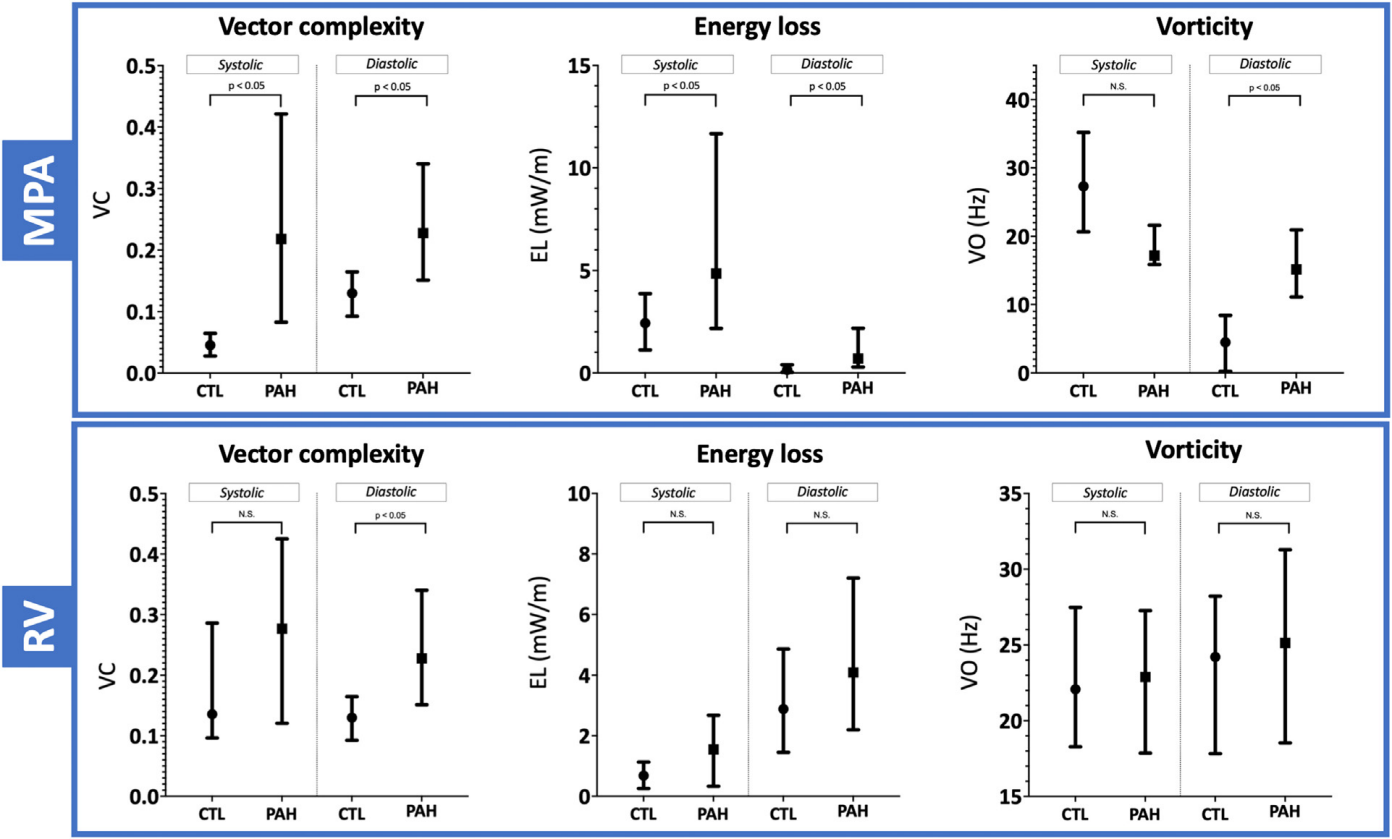
Quantitative flow parameters in the main pulmonary artery (MPA) and right ventricle (RV). Values expressed as median, quartiles (Q1-Q3). CTL, control; EL, energy loss; N.S., not significant; PAH, pulmonary arterial hypertension; VC, vector complexity; VO, vorticity.

Quantitative flow parameters are shown in Figure 3. The rate of EL in the MPA was higher in PAH in systole (4.84 vs 2.42 mW/m) and diastole (0.69 vs 0.14 mW/m). Vector complexity was also higher in systole (0.21 vs 0.04) and diastole (0.13 vs 0.05) in PAH compared with CTL. Vector complexity did not correlate with MPA z-score (r = 0.37, *P* = 0.15). Diastolic VO was higher in PAH compared with CTL (15.2 vs 4.4 Hz). In the RV, diastolic VC was higher in PAH compared with CTL, but RV EL and RV VO were not statistically significant between the groups.

The vortex duration did not correlate significantly with RV functional parameters (tricuspid annular systolic excursion: r = 0.22, *P* = 0.40; right ventricular fractional area change: r = −0.45, *P* = 0.11), with right ventricular systolic pressure (r = −0.01, *P* = 0.11) or pulmonary artery dimensions (RPA_D_: r = 0.17, *P* = 0.51; MPA z-score: r = −0.21, *P* = 0.41). MPA EL did not correlate with any RV functional parameters or any RV flow parameters (RV EL, VO, and VC). The quantitative flow parameters in the MPA and RV did not correlate significantly with right ventricular systolic pressure.

**Figure 4:**
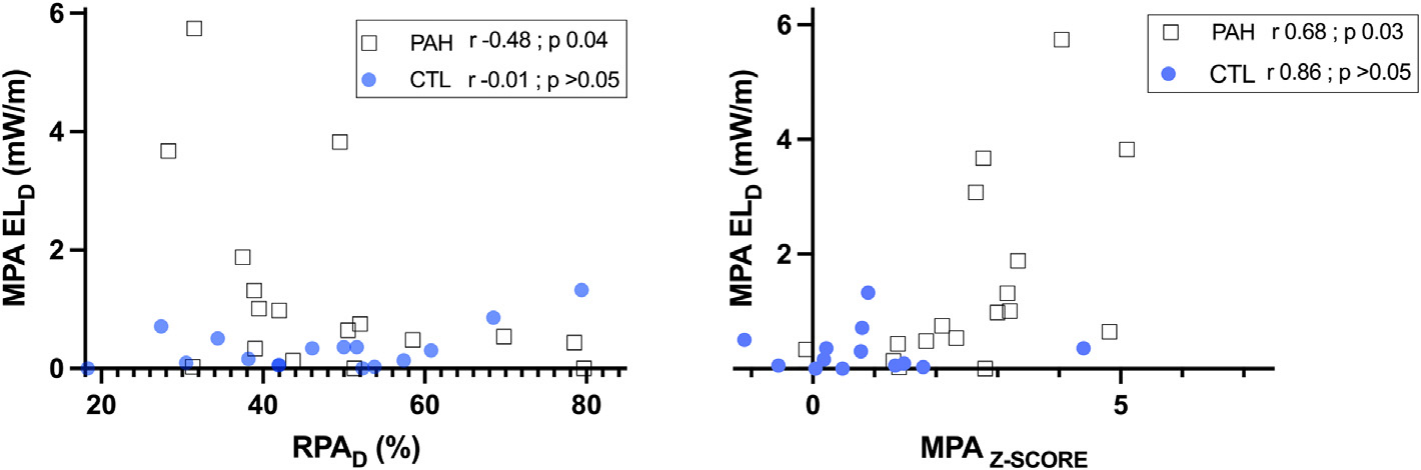
Correlation of main pulmonary artery (MPA) diastolic energy loss (EL_D_) with right pulmonary artery distensibility (RPA_D_) index and MPA z-score. CTL, controls; PAH, pulmonary arterial hypertension.

When comparing the RPA_D_ index (Fig. 1), no significant differences were noted between the groups (CTL: 23.9% vs PAH: 24.15%). In patients with PAH, diastolic MPA EL negatively correlated with RPA_D_ (r = −0.48; *P* = 0.04) and positively correlated with MPA z-score (r = 0.68; *P* = 0.03) (Fig. 4).

## Discussion

Using high-frame-rate BST echocardiography, our data demonstrate important qualitative and quantitative differences in flow dynamics, particularly in the MPA in children with PAH compared with CTL. Interestingly, in the RV, the only difference noted was vector complexity in diastole, with no differences in systole.

The most important finding of our study was the presence of an MPA diastolic vortex in most patients with PAH, which is a distinguishing flow feature in patients with PAH. Abnormal diastolic flow in the MPA and in the pulmonary branches can often be observed using conventional color Doppler and is thought to represent reflection of pulmonary artery flow related to high distal resistance. The advantage of BST technology is that it allows better visualization of the vortices and it allows quantification of flow parameters. In cardiac MRI studies, it was previously suggested that diastolic vortex duration correlated with pulmonary artery pressures,^11^ but this could not be demonstrated in our study. A possible explanation is the higher temporal resolution of BST compared with MRI with the echocardiographic data likely to be more physiological. Alternatively, the difference could potentially be explained by the 2-dimensional nature of our echocardiographic acquisitions, which may not capture through-plane motion of the vortices, thus leading to an underestimation of their duration compared with a 3-dimensional imaging modality such as MRI. It was interesting to observe that, even in patients’ milder PAH with RV systolic pressure less than half-systemic, a diastolic MPA vortex could be observed. Although this study could not validate the value of vortex duration as a noninvasive marker of PAH severity, the use of vortex detection as a diagnostic sign for PAH requires further validation. Some ventricular flow disturbances have been shown to precede geometric remodelling.^12^ If this also applies to MPA flow, the value of these novel flow parameters is promising although this study is not geared to demonstrate this.

When quantifying flow parameters, we could find important differences between the groups, especially in the MPA with higher MPA EL and higher VC in patients with PAH vs CTL. The higher EL difference could potentially be explained by a loss of kinetic energy in the blood flow in the form of thermal energy because of friction forces related to the more complex flow patterns in the MPA. Our data demonstrate that the MPA flow was more complex with wider spread of vector directions, suggested by the higher vector complexity. The vortices in the MPA differ from an energetic perspective from the ones observed in the ventricles where they often serve as conservers of kinetic energy from the diastolic to systolic phase. The diastolic vortex observed in most PAH contributes to increased EL contributing to inefficiencies in the pulmonary arterial circulation.

Vector complexity, which is a flow parameter reflecting the spread of the direction of the velocity vector fields,^9^ shows significantly higher values in the MPA and RV of the PAH group compared with CTL. This reflects the less laminar flow occurring in PAH both in systole and diastole. Although we did not observe decreased VO in systole as reported previously,^3^ VC did show differences between the groups. This is perhaps because the VC parameter is more reflective of the nonlaminarity of flow and less depended on velocities as would EL and VO. This flow metric could also illustrate the systolic-diastolic coupling of flow parameters, where a less laminar, rotating diastolic vortex in the MPA will lead to less laminar flow in systole as well.

In PAH, MPA diastolic EL correlated negatively with RPA_D_ and positively with MPA z-score, whereas no such correlations were found in CTL. These findings are in favour of increased vascular stiffness as a more dilated and less distensible pulmonary arterial system in PAH provides less elastic recoil to continue propelling blood forward in diastole and predisposes it to circular, more complex flow patterns with higher energy losses. Although the literature is sparse, the role of such a measurement has been shown to be valuable in humans^13^, ^14^, ^15^, ^16^ and dogs^17^^,^^18^ with PAH as an additional noninvasive marker of pulmonary arterial stiffness. The combination of blood flow quantification with noninvasive markers of pulmonary arterial stiffness gives more insights into the particularities of the RV-PA unit function in PAH.

### Study limitations

There are several limitations to this study. The first is the small sample size. The availability of the BST imaging technique only for higher frequency probes precluded inclusion of older and bigger children, which limited recruitment. The study also suffers from the lack of contemporaneous invasive measurements of PVR, MPA pressures to relate the flow parameters to. In addition, this study is not designed to test clinical uses of qualitative and quantitative flow parameters but rather to use this technology to describe flow disturbances in PAH compared with CTL. This study should be considered a pilot, hypothesis generating and feasibility study using a novel imaging technology. Another limitation is the 2-dimensional nature of the imaging technique, thus not accounting for through-plane variations. EL calculations are dependent on smoothing parameters.^8^ We used the same parameters across our analysis to avoid this potential source of error.

## Conclusion

The use of high-frame-rate ultrasound imaging allows us to demonstrate abnormal flow characteristics in the MPA and RV in patients with pulmonary hypertension compared with CTL. An abnormal MPA diastolic vortex can be identified in most patients with PAH, even in those with milder disease severity compared with CTL. Even as right ventricular EL and VO did not differ significantly, VC was higher in PAH compared with CTL. Higher energy losses and vector complexity in the MPA can result from increased right ventricular afterload due to increased PVR as well as increased MPA stiffness. These novel flow parameters are promising noninvasive markers of disease severity, and further investigation is warranted.
